# Functional stirred yogurt manufactured using co‐microencapsulated or free forms of grape pomace and flaxseed oil as bioactive ingredients: Physicochemical, antioxidant, rheological, microstructural, and sensory properties

**DOI:** 10.1002/fsn3.3385

**Published:** 2023-04-21

**Authors:** Manaf Saberi, Solmaz Saremnezhad, Mostafa Soltani, Alireza Faraji

**Affiliations:** ^1^ Department of Food Sciences and Technology, Faculty of Pharmacy, Tehran Medical Sciences Islamic Azad University Tehran Iran; ^2^ Nutrition and Food Sciences Research Center, Tehran Medical Sciences Islamic Azad University Tehran Iran; ^3^ Department of Organic Chemistry, Faculty of Pharmaceutical Chemistry, Tehran Medical Sciences Islamic Azad University Tehran Iran

**Keywords:** antioxidant activity, flaxseed oil, functional stirred yogurt, grape pomace, microencapsulation, rheological properties

## Abstract

Functional stirred yogurt samples were manufactured with combinations of grape pomace (GP) and flaxseed oil (FO) in microencapsulated or free forms (2% w/w) and quality characteristics of yogurts were investigated during 21 days of storage. The incorporation of GP and FO in microencapsulated or free forms caused a significant decrease in pH, syneresis, and a significant increase in acidity, water holding capacity, and viscosity of stirred yogurt (*p* < .05). While stirred yogurt containing GP and FO in free form had the highest loss modulus (G″), all yogurt samples represented solid‐like behavior. Stirred yogurts containing the microencapsulated form of GP and FO showed the highest amount of phenolics and antioxidant activity compared with the two other yogurt samples (*p* < .05). More compact structure and higher gel strength were observed in stirred yogurts formulated with the microencapsulated or free form of GP and FO, compared to the control yogurt sample. The overall sensory acceptability of stirred yogurt manufactured using the encapsulated form of GP and FO was not significantly different from the control yogurt sample (*p* > .05). In conclusion of this competitive study, GP and FO as bioactive compounds could be used in the microencapsulated form in order to develop functional stirred yogurt with specific quality characteristics.

## Graphical abstract

Stirred yogurt was fortified with two bioactive ingredients including grape pomace and flaxseed oil to achieve health benefits. Microencapsulated yogurt had higher antioxidant activity compared to the control one. The sensory characteristics of microencapsulated yogurt were acceptable.

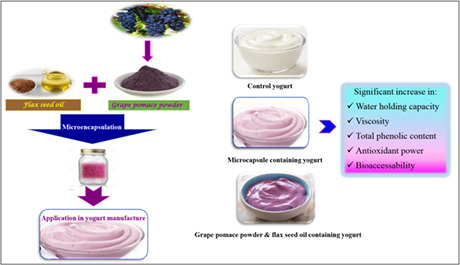

## 1 INTRODUCTION

Grape (*Vitis vinifera* L.) is a rich source of dietary fiber and phenolic compounds with high antioxidant activity. Grape pomace (GP) is usually referred to a by‐product including pulp, seeds, and peels of fruit and known as a rich source of phenolic compounds, that is, epicatechin, catechin, gallic acid, procyanidins, and phenolic acids. The presence of these compounds leads to existing important biological activities such as antioxidant and antimicrobial activities in the food products containing grape pomace (Andrade et al., 2019; Caldas et al., 2018; Pourali et al., 2014).

Flaxseed oil (FO) is obtained from the seed of the flax plant (*Linum usitatissimum* L.), and contains an essential omega‐3 fatty acid called alpha‐linolenic acid, and phenolic compounds (Kaushik et al., 2016). Several studies have declared the health benefits of foods fortified with grape by‐products and flaxseed oil (Almasi et al., 2021; Baba et al., 2018; Kaushik et al., 2016; Varedesara et al., 2021; Yadav et al., 2018).

The quality characteristics of the food products may be affected by the astringency and high‐intense purple color of grape used in formulation (Pourali et al., 2014). On the other hand, the high unsaturated fatty acid content of flaxseed oil makes it extremely sensitive to oxidation reactions and may cause less biological functionality in formulated food. For resolving these issues, microencapsulation of mentioned unstable compounds is proposed by various studies (Kaushik et al., 2016). Microencapsulation is a coating process that protects core material, that is, solid particles, gas components, and liquids by a wall material. This is a promising technology for stabilization of active components (mostly expensive and sensitive nutrients) in food, protecting them from other reactive components and subsequently, releasing at a specific time and dose and under specific conditions. In addition, utilization of microencapsulation can protect or enhance the sensory quality of food products by masking the unpleasant taste and aroma (Choudhury et al., 2021). In this regard, selection of the wall material is a crucial step of microencapsulation. The wall material should be tasteless, flexible, nonhygroscopic, soluble in different solvents, and have a film‐forming capability, as well as inert on the core materials and cost‐effective (Li et al., 2021). Previous studies have successfully used different types of natural biopolymers including maltodextrin and gum tragacanth as wall materials in microencapsulation. Maltodextrins are a class of carbohydrates that act as a good microencapsulating agent due to their high solubility, low viscosity, and drying properties. Gum tragacanth is a natural odorless and tasteless gum which is stable at a pH range of 4–8 and can be used as stabilizer in food formulations (Hofman et al., 2016; Kaushik et al., 2016).

Nowadays, the demand for functional foods with health‐enhancing and nutrition‐modified properties has constantly raised due to the increase in consumer's awareness and concern about health aspects of food products (Kandylis et al., 2021; Matos et al., 2021). The collaboration of sciences and consumer demand is eventuated to functional foods (Pourali et al., 2014). Functional foods are defined as all foods that offer physiological benefits due to the presence of physiologically active substances.

Yogurt, an acidified fermented dairy product provides nutritional benefits and boosts the immune and digestive system (Matos et al., 2021; Say et al., 2019). The potential of yogurt as a proper functional food for conferring health benefits has also been proved (Ahmad et al., 2022; Ahmed et al., 2021). The aim of the present study was to evaluate the effect of the combination of GP and FO in free and microencapsulated form (by maltodextrine and gum tragacanth) on the quality characteristics of yogurt during 21 days of storage.

## 2 MATERIALS AND METHODS

Grape (*vitis vinifera* L. *Khashnav*) was prepared from the Agricultural Research, Education and Extension Organization, West Azerbaijan province, Iran. Flaxseed oil was provided from local market. Iranian gum tragacanth (*Astragalus gossypinus*, Fars province, Iran) grounded and passed through a 40‐mesh sieve before using for preparation of samples. Skim milk powder and lyophilized yogurt starter culture (1:1 ratio, code: YC 350, Yo‐Flex) containing *Streptococcus thermophilus* and *Lactobacillus delbruckii* ssp. *bulgaricus* were provided from Pegah Dairy Co. and CHR‐Hansen Company, respectively. Chemicals used during analysis were all analytical grade and purchased from Merck Company.

### 2.1 Preparation of grape pomace powder

To prepare pomace, grapes were crushed and juiced. Then, the obtained pomace was freeze‐dried in a LYOTRAP freeze dryer (LTE Scientific Ltd) and grinded in a Quadro Comil grinder (Model 197, Quadro Engineering Corp) and sieved by a 475 μm sieve. The dry powder was stored at −20°C.

### 2.2 Microencapsulation procedure

The wall materials including maltodextrin and gum tragacanth were mixed together at a certain ratio (1/1 w/w). Citric acid and leucine amino acid were used for crosslinking between wall materials and removing hydrogen ions (H^+^) in order to create a partial hydrophobic nature in microcapsule, respectively. The crosslinking reactions occurs between hydroxyl groups of maltodextrin and gum tragacanth and carboxylic groups of citric acid (Francisco et al., 2018).

The preparation of microcapsule followed the procedure reported by da Silva et al. (2019), with some modifications. Combination of GP with FO (1:1 w/w) gently mixed with prepared wall material. The mixture was then dried using spray‐drying technique. The drying process was carried out using Büchi B‐290 mini spray drier with double fluid nozzle atomizer of 0.7 mm diameter. The conditions used for drying operation were 40 m^3^/h air flow rate; 140°C ± 2°C for inlet air temperature; 70°C ± 2°C for outlet air temperature; and 5 mL/min for feed flow rate. The formed microcapsules were collected at the bottom of the drier. The obtained microcapsules were kept in aluminum sealed bags and stored in −18°C until use.

### 2.3 Microencapsulation yield

The ratio between the weight of obtained microcapsules and the weight of encapsulation materials (wall and core materials) was calculated as the yield of the microencapsulation process (Ribeiro et al., 2015).

### 2.4 Yogurt manufacture

The raw cow milk was fat standardized (3.0%) and then concentrated with adding 2.0% of skim milk powder (at 45°C) for increasing the total solid content and improving the consistency of final product. Next, the milk was heat treated to 90°C for 5 min, cooled to 44°C ± 0.05°C, inoculated with 3% (v/v) of yogurt starter culture and incubated at 42°C ± 0.05 until dropping pH to isoelectric point (pH 4.6) and formation of the curd. At the next stage, three batches of stirred yogurt treatments (including control yogurt) were prepared by adding 2% w/w of combination of GP (1% w/w) and FO (1% w/w) in microencapsulated and free forms and coded as given in Table 1. All three batches were then dispensed into plastic containers (200 mL) and stored at 4°C ± 0.01°C for 21 days. The day after manufacturing was calculated as the first day of storage and analyses were carried out in triplicate on 1, 7, 14 and 21 days of cold storage.

**TABLE 1:** Different yogurts manufactured in the present study.

Code	Treatment
CY	Control yogurt
MGFY	Yogurt manufactured with combination of grape pomace (1% w/w) and flaxseed oil (1% w/w) in microencapsulated form
FGFY	Yogurt manufactured with combination of grape pomace (1% w/w) and flaxseed oil (1% w/w) in free form

### 2.5 Physicochemical analysis

The pH of the stirred yogurt samples was measured using digital pH meter (WTW). The titratable acidity was determined by alkali (0.1 N NaOH) titration method. Total solid content was determined by oven‐drying method at 100°C ± 1°C. (AOAC, 2016). The fat and protein contents were analyzed according to Gerber method described by Zhao et al. (2020) and total nitrogen method of Kjeldahl (AOAC, 2016), respectively. For determination of whey separation, a filter paper (no. 589/2, 0.00009 g) placed on the top of a funnel and samples were weighed (25 g) on this. The final value was expressed as the percent of the amount of drained liquid (g) during 120 min at 4°C, per 25 g of sample (Al‐Kadamany et al., 2003). The water holding capacity of yogurt samples was determined according to the method described by Dai et al. (2016). Five grams of yogurt samples were centrifuged at 2264 *g* for 30 min at 4°C. The supernatant was collected and weighted. The WHC was calculated according to the Equation 1.

(1) $$\text{Water holding capacity}\;\left(\%\right)=\left(1-\frac{W}{W^{’}}\right)\times 100$$

where, *W* is the weight of the supernatant and $W^{\prime }$ is the initial weight of the sample.

### 2.6 Rheological analysis

Rheological properties of the yogurt samples were measured using a rheometer (Anton Paar Germany GmbH) fitted with a cone and plate geometry (50 mm diameter and 2° of inclination angle). Samples were loaded and spread on the surface of the horizontal plate and excess samples were trimmed off. A displacement of 0.002 rad was chosen for the frequency sweep to be tested at 0.01–1 Hz. The elastic modulus (G′), and loss modulus (G″) as primary rheological terms were monitored during the analysis (Mudgil et al., 2018).

### 2.7 Microstructure

The microstructure of the yogurt samples was screened using scanning electron microscopy (SEM) as described by Zhao et al. (2020). For this purpose, yogurt sample (about 5 μg) was layered gently on a silica plate, and frozen in liquid nitrogen, subsequently lyophilized using freeze dryer. The lyophilized powder, after coating with a thin layer (15 nm) of gold–palladium (Model SC7620; Quorum Technologies), was mounted on the aluminum stub of SEM (JSM‐7001F; JEOL) and scanned. SEM was operated at 30 kV (electron accelerating voltage), photomicrographs were recorded under 100 to 5000 × magnified images and structural differences were analyzed in 5000 × times magnified images.

### 2.8 Fourier‐transform infrared spectroscopy (FTIR) analysis

FTIR analysis (Nicolet, Thermo Electron) was performed according to the method described by Mudgil et al. (2018). Yogurt samples were freeze‐dried and the lyophilized powder mixed thoroughly with potassium bromide (KBr), then pressed enough to transform into small, transparent disks. The infrared spectra of samples were collected at a resolution of 4 cm^−1^ in the range of 4000–650 cm^−1^.

### 2.9 Total phenolic contents (TPCs) and antioxidant activity

The TPC of yogurt samples was determined using the Folin–Ciocalteu's phenol method, (Ahmed et al., 2021). First, 100 g of yogurt samples was centrifuged at 5000 × g for 5 min at 4°C and then, re‐centrifugation of the supernatants was implemented in the same conditions. Next, a mix of 100 μL of yogurt extract with an equal volume of Folin–Ciocalteu's solution (1 mol/L) was prepared and 300 μL of 1 mol/L sodium carbonate solution was added to the mix after a 5 min incubation at room temperature. The obtained solution kept at room temperature for 30 min. At the final stage, 1 mL distilled water was added to the mixture and the absorbance was recorded at 725 nm (Lambda EZ 150). The results were reported as mg of GAE per 100 g of each yogurt sample.

Determination of DPPH (2,2‐Diphenyl‐1‐picrylhydrazyl) radical scavenging activity was used for assessment of antioxidant activity of yogurt samples (Demirci et al., 2017). Briefly, 100 μL of extraction sample was mixed with 2 mL of diluted DPPH radicals. The obtained mix was then kept for 30 min in dark (at room temperature). The absorbance was measured at 517 nm (Lambda EZ 150), and the results were given as inhibition percent using the following equation:

$$\text{Inhibition}\;\left(\%\right)=\left(A_{\text{control}\;\left(517\right)}–A_{\text{extract}\;\left(517\right)}/A_{\text{control}\;\left(517\right)}\right)\times 100$$

### 2.10 Release characteristic

To investigate the in vitro release behavior of microcapsules, both simulated gastric fluid (SGF) and simulated intestinal fluid (SIF) model were prepared according to the method reported by Wang et al. (2018). To prepare SGF solution, 2 g of NaCl and 7.0 mL of 36% diluted hydrochloric acid were dissolved in 900 mL deionized water and mixed with 3.2 g pepsin enzyme. The pH of the solution was maintained at 1.2 using HCl (36%). The solution made up to 1000 mL by adding deionized water. In order to prepare SIF solution, 6.8 g KH_2_PO_4_ was mixed in 77 mL of 0.2 M NaOH and dissolved in 800 mL deionized water. The pH of the solution was adjusted to 6.8 using 1 M NaOH or 1 M HCl. The volume of solution was made up to 1000 mL with distilled water followed by adding 10 g pancreatin to the mixture. The obtained solutions should be used instantly to prevent enzyme inactivation. In the next step, 2 g of sample was dissolved in the prepared SGF solution (20 mL) and incubated for 2 h at 37 ± 0.5°C with constant agitation at 100 rpm in water bath for better simulation. After 2 h, the pH was instantly adjusted to 6.8 by adding NaOH (1 M), in order to inactivate pepsin. Then the mixture was digested sequentially in 20 mL SIF solution under the same conditions for 3 h. Finally, the solutions were centrifuged at 6238 g for 5 min, the supernatant was filtered through a membrane filter (0.22 μm) and the TPC and antioxidant activity were measured as described in 2.9 section.

### 2.11 Sensory evaluation

The sensory properties of the yogurt samples were carried out using a 5‐point hedonic scale (1: unacceptable; 2: somewhat acceptable; 3: acceptable; 4: desirable; 5: excellent). Eleven panelists evaluated sensory quality of samples including appearance and color, consistency (perceived by mouth or spoon), odor and flavor, and overall acceptability. Samples with three digit codes were removed from the refrigerator and presented to each panelist, randomly (Brennan & Tudorica, 2008). Water and bread were provided for panelists in order to cleanse palates between samples.

### 2.12 Statistical analysis

The analysis of variance (ANOVA) of SPSS statistics software (SPSS package program, version 22.0, SPSS Inc.) was used for analysis of data obtained from three trials. Duncan's multiple range tests were applied for determination the differences among treatments. Analysis was performed for 1, 7, 14, and 21 days of storage. The results were considered significant at *α* = .05.

## 3 RESULTS AND DISCUSSION

### 3.1 Microencapsulation yield

The yield calculated for microencapsulation of GP and FO was 76.54% ± 0.37% indicating that an effective encapsulation procedure has been resulted using maltodextrin and gum tragacanth, as wall materials. The creation of hydrogen bonds between carboxylic groups of wall materials could help to existing remarkable encapsulating properties for maltodextrin and gum tragacanth (da Silva et al., 2019; Khorshidi et al., 2021).

### 3.2 Chemical composition of milk and yogurts

The composition of milk used for manufacturing of yogurt samples was determined as 12.23% ± 0.08% for total solid, 3.10% ± 0.00% for fat, 3.26% ± 0.04% for protein, 6.73% ± 0.02 for pH, and 0.15% ± 0.00% of lactic acid for acidity. The total solid, fat, and protein contents of yogurts at the first day of storage are shown in Table 2.

**TABLE 2:** Chemical composition of yogurts at the first day of storage.

Yogurts	Total solid (%)	Fat (%)	Protein (%)
CY	15.66 ± 0.04^b^	3.13 ± 0.01^c^	4.26 ± 0.03^c^
MGFY	17.59 ± 0.11^a^	3.86 ± 0.02^b^	5.55 ± 0.07^b^
FGFY	18.07 ± 0.09^a^	4.06 ± 0.02^a^	6.28 ± 0.05^a^

*Note*: Yogurts codes are shown in Table 1. Analyses were performed in triplicate. Values are means ± SD. Small letters indicate a significant difference in the columns (the difference between the samples in a day of storage) at level of 5%.

As expected, the contents of total solid, fat, and protein in the yogurts manufactured with incorporation of encapsulated or free forms of grape pomace and flaxseed oil were significantly higher than control yogurt due to the high level of dietary fiber and protein content in grape pomace and the richness of the flaxseed oil in fatty acids (Cheng et al., 2019; Tang et al., 2018). In a related study, Yadav et al. (2018) reported an increase in the total solid and protein content of yogurt incorporated with grape seed extract compared to control one. Also, similar results about the increase in the fat, protein, and ash content of yogurt manufactured by incorporating of encapsulated echium oil was published by Comunian et al. (2017).

### 3.3 pH, titratable acidity, and physical properties

The values for pH, titratable acidity, whey separation, water holding capacity, and viscosity of yogurt samples are presented in Table 3. pH is a critical parameter in yogurt for maintaining the structure of microcapsule during storage. In this context, it was reported that pH in the range of 4 is ideal to maintain the microcapsule structure and prevent the release of encapsulated materials during yogurt storage (Comunian et al., 2017). The pH value ranged from 4.54 to 4.25 for MGFY during the storage period, which was in the optimum pH range to maintain the microcapsule structure. In accordance with this result, Pinto et al. (2019) reported 4.68 to 4.33 for pH value of probiotic yogurt fortified with microencapsulated *Bifidobacterium lactis* BB‐12, through 30 days of storage.

**TABLE 3:** pH, titratable acidity, and physical properties of yogurts during storage.

	Yogurts	1st day	7th day	14th day	21st day
pH	CY	4.57 ± 0.01^aA^	4.52 ± 0.01^aB^	4.38 ± 0.02^aC^	4.29 ± 0.01^aD^
	MGFY	4.54 ± 0.01^bA^	4.49 ± 0.02^bB^	4.34 ± 0.02^bC^	4.25 ± 0.01^bD^
	FGFY	4.51 ± 0.01^cA^	4.44 ± 0.01^cB^	4.29 ± 0.02^cC^	4.19 ± 0.02^cD^
Acidity (Lactic acid %)	CY	0.86 ± 0.02^cC^	0.97 ± 0.01^cB^	1.01 ± 0.02^cAB^	1.07 ± 0.01^cA^
	MGFY	1.11 ± 0.03^bC^	1.22 ± 0.01^bC^	1.17 ± 0.01^bB^	1.23 ± 0.03^bA^
	FGFY	1.21 ± 0.03^aC^	1.28 ± 0.01^aB^	1.32 ± 0.01^aB^	1.39 ± 0.02^aA^
Syneresis (g/25 g)	CY	3.81 ± 0.09^aD^	4.10 ± 0.04^aC^	5.42 ± 0.09^aB^	9.31 ± 0.04^aA^
	MGFY	2.92 ± 0.04^bD^	3.56 ± 0.03^bC^	4.62 ± 0.05^bB^	7.11 ± 0.06^bA^
	FGFY	0.81 ± 0.06^cD^	1.12 ± 0.02^cC^	2.20 ± 0.10^cB^	2.71 ± 0.09^cA^
Water holding capacity (%)	CY	95.65 ± 0.05^cA^	94.43 ± 0.16^cB^	92.97 ± 0.17^cC^	86.99 ± 0.09^cD^
	MGFY	98.02 ± 0.07^bA^	97.66 ± 0.11^bA^	95.47 ± 0.13^bB^	92.62 ± 0.18^bC^
	FGFY	99.35 ± 0.05^aA^	98.82 ± 0.03^aB^	97.56 ± 0.08^aC^	97.11 ± 0.13^aD^
Viscosity (cP)	CY	5327 ± 6.00^cA^	5063 ± 12.50^cB^	4802 ± 15.50^cC^	4211 ± 11.50^cD^
	MGFY	5647 ± 20.50^bA^	5330 ± 3.00^bB^	5146 ± 24.50^bC^	4764 ± 7.50^bD^
	FGFY	5985 ± 6.50^aA^	5706 ± 25.00^aB^	5383 ± 4.00^aC^	5114 ± 3.00^aD^

*Note*: Yogurts codes are shown in Table 1. Analyses were performed in triplicate. Values are means ± SD. Small and capital letters indicate a significant difference in the columns (the difference between the samples in a day of storage) and the rows (difference of one sample during storage) at level of 5%.

According to Table 3, the pH values in both fortified yogurt samples (MGFY and FGFY) were significantly less than control sample, during storage. In contrast, significant increase was seen in the titratable acidity of MGFY and FGFY compared to control yogurt (*p* < .05). It was reported that growth and activity of starter cultures and probiotic bacteria can be promoted by the presence of grape derivatives due to advantageous effect of some ingredients like organic acids, carbohydrates, phenolic compounds, and fibers (Kandylis et al., 2021). Decrease in pH and increase in titratable acidity of fortified yogurts might be occurred due to increasing the metabolic activity of lactic acid bacteria and production of organic acids by them (Moghadam et al., 2021). On the other hand, increase in the metabolic activity of lactic acid bacteria and production of lactic acid during fermentation of lactose by lactic acid bacteria might be the main reason for significant increase in titratable acidity (*p* < .05) and significant decrease in pH (*p* < .05) of yogurt samples during cold storage (Comunian et al., 2017; Yadav et al., 2018).

Syneresis or whey separation is occurred due to deformation and weakening of three‐dimensional network in yogurt gel and loss of the yogurt gel ability to retain the serum phase. This phenomenon is considered as one of the most critical parameters in determining the quality of yogurt samples during storage period. Based on the obtained results, using combination of GP and FO in microencapsulated or free forms significantly decreased the value of syneresis in yogurt (*p* < .05). The highest syneresis value was related to the CY on 21st day of storage (9.31 ± 0.04), while FGFY had the lowest syneresis value (0.81 ± 0.06) at the first day of storage. Formation of stable complexes by multiple hydrophobic interactions between aromatic rings of polyphenols and amino acid side chains of proteins could reduce the syneresis value in the yogurts fortified with combination of GP and FO. In addition, increasing the total solid content can incorporate in the reduction of syneresis by restraining the free water (Vital et al., 2015). Syneresis was significantly increased during storage in all samples, which may be related to the deformation and contraction of the casein matrix that causes the reduction in ability of caseins for retaining the serum phase and losing the stability of the yogurt structure (Kokabi et al., 2021; Vital et al., 2015).

The water holding capacity (WHC) is identified as the capability of foods to hold all or a part of water (Pourali et al., 2014). According to the results shown in Table 3, WHC of yogurt samples was depended on the type of fortification (encapsulated or un‐capsulated) and storage time. The WHC value in FGFY was significantly higher than other samples during storage, due to the presence of dietary fiber in grape residue extract (Kandylis et al., 2021). On the other hand, The WHC of all samples was significantly decreased during storage time (*p* < .05), which may be related to the decrease in the entrapment of solids into the protein matrix and reducing the stability of yogurt matrix as a result of rearrangement of the gel network (Bakry et al., 2019).

The viscosity values of the yogurt samples during 21 days of cold storage significantly (*p* < .05) affected by incorporation of GP and FO, in microencapsulated or free forms (Table 3). Both fortified yogurt samples showed higher viscosity values compared to the control yogurt, which expressed the lowest viscosity during storage. Gum tragacanth used as wall material in MGFY is an anionic polymer. The polyphenols in FGFY are also anionic polyelectrolytes. Therefore, these compounds with anionic properties can interact with positive charge particles within yogurt matrix consisting of calcium ions and/or proteins. These interactions could be resulted in an increase in the viscosity of the yogurt gel matrix through the strengthening of the protein network (Khorshidi et al., 2021).

### 3.4 Rheological properties

Various parameters, that is, method of manufacturing, type of starter cultures, conditions of heat treatment and composition of final product can influence the rheological properties of the food (Jaster et al., 2018).

**FIGURE 1:**
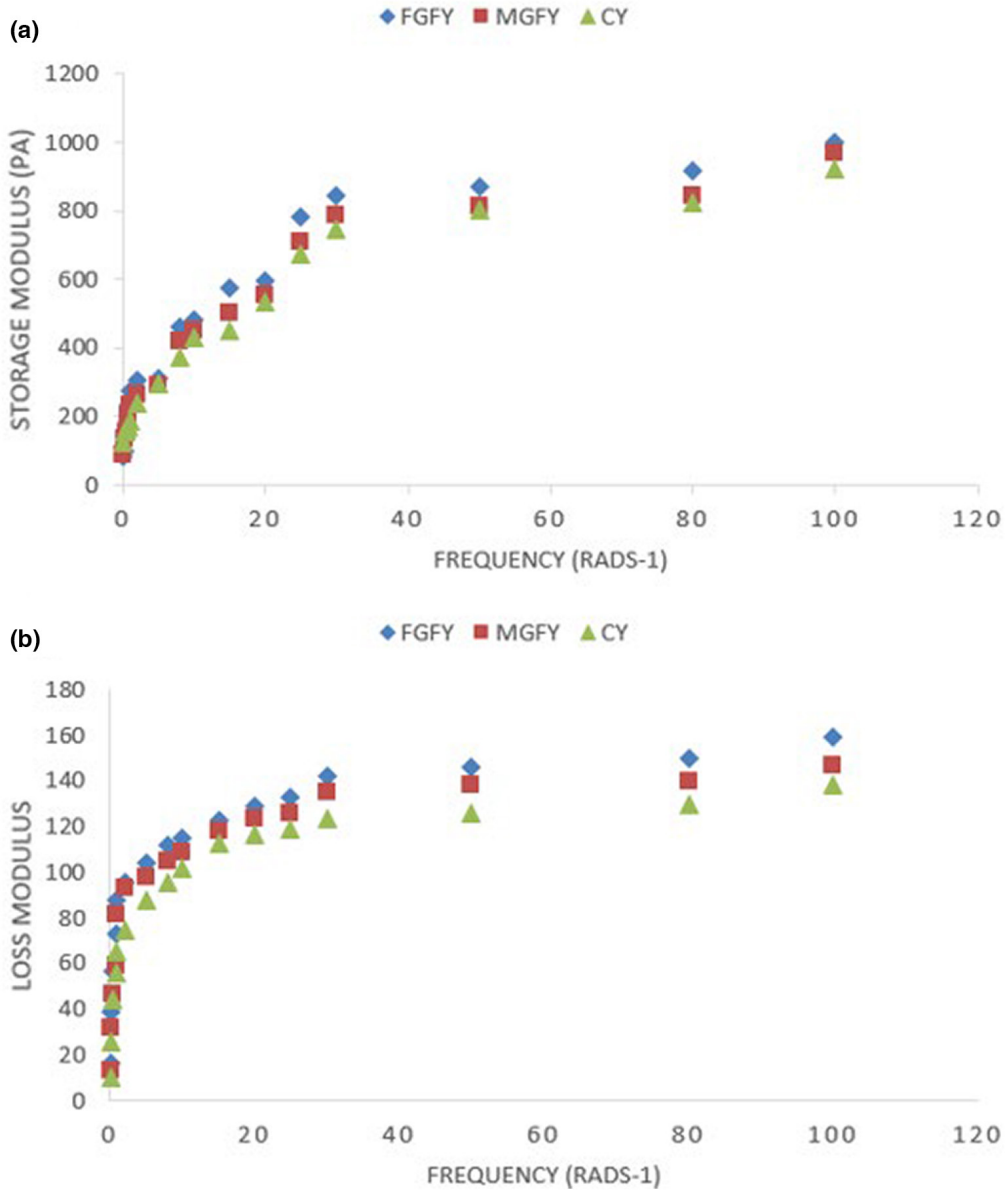
Rheological properties of yogurt samples including storage modulus (a) and loss modulus (b) at the first day of storage. Yogurts codes are shown in Table 1.

Storage modulus and loss modulus are two main factors for measurement the rheology characteristics of food systems. The storage modulus (G′) is defined as the elastic energy of a food sample generated by deformation and used for measurement of the elasticity in a food. On the other hand, the loss modulus (G″) is defined as the loss of energy in a food sample and is used for the measurement of viscosity characteristics in a food (Li et al., 2021). As shown in Figure 1, with increase in the frequency, the G′ and G″ of all yogurt samples increased. Furthermore, the value of G′ was higher than the value of G″ in yogurt samples during analyzed frequency range. These results can state that all three yogurt samples represented elastic behavior with a solid‐like rheological characteristics.

In terms of storage modulus (G′), both fortified yogurt samples had higher values compared with control yogurt. The highest storage modulus (G′) was observed in FGFY, probably due to creation of interactions between casein aggregates and dietary fibers of GP (Bakirci et al., 2017). Moreover, the association between wall materials of microcapsules (maltodextrin and gum tragacanth) and casein micelles via hydrogen bonding and dipole–dipole interactions caused to increase the storage modulus (G′) in FGFY compared with the control yogurt (Lima et al., 2021).

The loss modulus (G″) of MGFY and FGFY was higher than control yogurt. The materials used for microencapsulation of GP and FO, that is, maltodextrin and gum tragacanth can bond water molecules within yogurt matrix and improve the viscosity in MGFY (Kailasapathy, 2006). In a related study, Jouki et al. (2021), reported that contribution of microencapsulated *L*. *Plantarum* (in alginate‐skim milk microcapsules) improved viscosity characteristics of fresh yogurt due to increase in dry matter and water absorption property of wall materials of microcapsules. On the other hand, the highest loss modulus (G″) was observed in FGFY due to formation of super‐aggregates created among GP particles, FO droplets, and protein micelles within yogurt matrix. Moreover, hydration and swelling characteristics of GP particles dispersed in the yogurt matrix can significantly contribute to the increase in loss modulus (G″) of the product (Wang et al., 2019, 2020).

### 3.5 FTIR spectra

**FIGURE 2:**
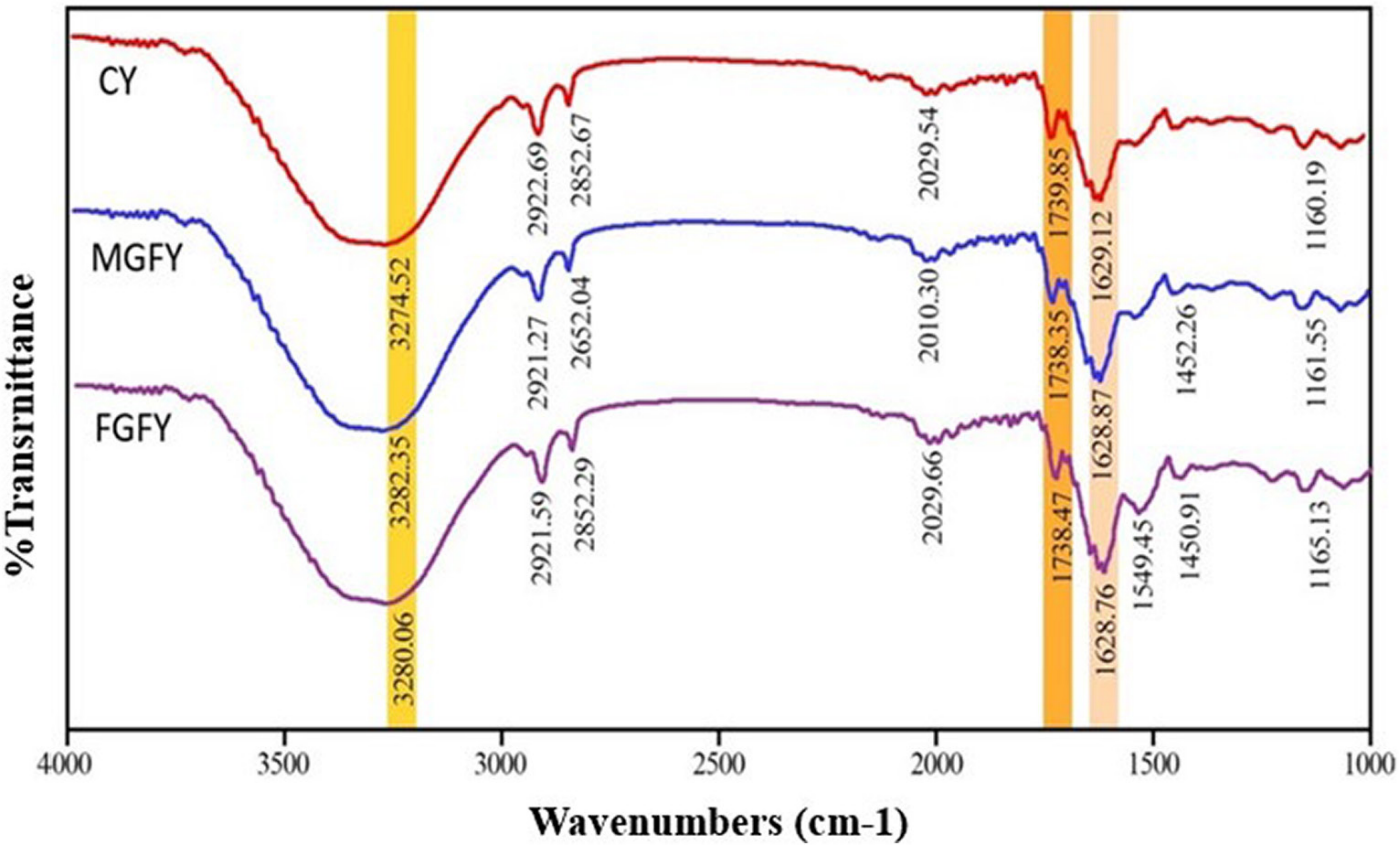
FTIR spectroscopy of yogurt samples at the first day of storage. Yogurts codes are shown in Table 1.

FTIR spectroscopy was used to monitor the changes in the yogurts structure before and after fortification and carried out for control and fortified stirred yogurt samples (see Figure 2). The main wavelengths related to the proteins, carbohydrates, and fats present in yogurt samples were observed. All yogurt samples showed a broad peak in the region 3250–3300 cm^−1^, which was related to stretching vibration of the hydroxyl group due to the amount of ‐OH groups in lactose (3250–3254 cm^−1^). The other observed peaks in all samples were 2921–2922 cm^−1^ which are related to the symmetric CH_2_ stretching of fatty acids; the peaks at 2852 cm^−1^ confirm the existence of methylene of lipids, and asymmetric ‐CH_2_ stretching mode of the methylene chains in membrane lipids (Santiago‐García et al., 2021). The major peaks at 1738–1739 cm^−1^ reflect C=O stretch of an ester or carboxylic acid function (Moros et al., 2006). Moreover, a band at 1628 cm^−1^ is centered on a region where amid I band and absorbed water are dominated, and it can be associated with O‐H, C=O, C‐N, and N‐H. Also, while the peak at 1549 cm^−1^ confirms the existence of amide II of proteins and ‐CH_2_ bending, the peaks at 1160–1165 cm^−1^ are attributed to fat C‐O stretch or carbohydrates C‐O stretch (Papadopoulou et al., 2021; Santiago‐García et al., 2021).

The most remarkable differences between IR spectral of yogurt samples were observed in the region 3250–3350 cm^−1^ and 1548 cm^−1^, which FGFY showed higher peaks in these regions than CY and MGFY. The comparison between yogurt samples spectra also displays a slight shift in the peaks at 1629 cm^−1^ and 1739 cm^−1^ to lower wavenumbers (1628 cm^−1^ and 1739 cm^−1^), which is related to C=C and C=O. Furthermore, the peak of NH_2_/OH (3274 cm^−1^) was shifted to higher wavenumber (3282 cm^−1^). These changes could be related to the electrostatic interactions between the yogurt and grape pomace–flaxseed oil combination.

### 3.6 Microstructure

**FIGURE 3:**
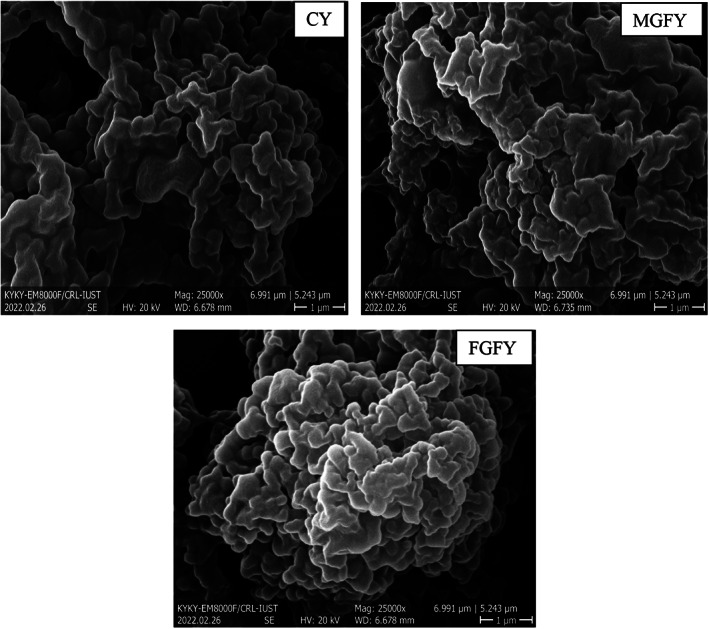
Microstructure of yogurt samples at the first day of storage. Yogurts codes are shown in Table 1.

Aggregation of casein micelles and denaturation of whey proteins during fermentation process results in the formation of a three‐dimensional network in yogurt. In this context, changes in formulation can lead to changes in gel microstructure of the product which can be visible using instruments like scanning electron microscope (Qu et al., 2021). SEM analysis performed in order to visualize the changes in the morphology of yogurt samples (Figure 3). Use of GP and FO in microencapsulated or free form in formulation of stirred yogurt led to some differences in the appearance of the gel network. According to the obtained micrographs, more compact and dense structure was observed in MGFY and FGFY compared to CY. Obviously, the size of network pores and aggregation pores progressively decreased in yogurts fortified with GP and FO in microencapsulated or free form which led to more viscose and tight structure with reduced dehydration and shrinkage. The polysaccharides used as wall material for formation of microcapsules had ability to bond with water molecules in the yogurt structure and could increase the consistency and stability of gel structure in MGFY (Bakry et al., 2019; Ladjevardi et al., 2015; Li et al., 2021).

On the other hand, the gel strength of FGFY was increased due to the incorporation of soluble sugars and pectin presented in GP in the stability of gel network structure. The interaction of polysaccharide–protein can enhance the stability of gel network in yogurt and lead to formation of more compact structure with small pores. It can be concluded that manufacture of stirred yogurt with microencapsulated or free forms of GP and FO can promote the formation of more continuous and compact gel network structure (Ning et al., 2021).

### 3.7 Total phenolic content (TPC) and antioxidant activity

Total phenolic content and antioxidant activity of the yogurt samples are shown in Table 4. Fortification of yogurt with GP and FO influenced the TPC of samples, as the values of TPC for fortified yogurts were significantly (*p* < .05) higher than control yogurt, during storage. These data revealed that the addition of GP and FO in microencapsulated or free form into the yogurt contributed significantly to the enhancement of the phenolic content. GP is a rich source of phenolic compounds, that is, epicatechin, catechin, gallic acid, procyanidins, and phenolic acids. These compounds have stable and powerful structures and are placed in the category of dietary antioxidants like carotenoids and vitamins (Yadav et al., 2018). Therefore, fortification of yogurt milk with GP before fermentation can increase the TPC of the final product. In this context, Akca and Akpinar (2021), also stated that addition of fruit‐based waste products (like grape pomace) and the oil of plant seeds (like flaxseed oils) can improve the functional properties of dairy products (such as yogurt), which are poor in phenolic and antioxidant compounds compared to the food products manufactured from plant derivatives. According to the results, the TPC in MGFY was significantly higher than FGFY (*p* < .05), during storage due to the protection of phenolic and antioxidant compounds from destructive parameters such as pH, oxygen, light, etc. in the microcapsules (Moghadam et al., 2021). On the other hand, it was observed that during storage, the TPC was decreased in all yogurt samples, significantly (*p* < .05) and reached to the lowest value at the 21st day of storage. It seems that the metabolic activity of yogurt starters, prolonged fermentation (21 days), and pH reduction can result in partial degradation of microcapsules and consumption and/or degradation of the microcapsules' core materials and result in the reduction of TPC in all yogurt samples during storage. Confirmatory results are reported by Muniandy et al. (2016) for yogurt fortified by white and black tea and Yadav et al. (2018) for yogurt manufactured with encapsulated grape seed extract.

**TABLE 4:** Total phenolic content and antioxidant activity of yogurts at 1st and 21st days of storage.

	Yogurts	1st day	21st day
Total phenolic content (mg GAE/g)	CY	71.03 ± 0.12^cA^	42.47 ± 0.05^cB^
	MGFY	134.26 ± 0.13^aA^	114.71 ± 0.11^aB^
	FGFY	105.72 ± 0.11^bA^	103.58 ± 0.08^bB^
DPPH radical scavenging activity (IC50%, mg/ml)	CY	163.64 ± 0.01^aA^	154.65 ± 0.01^aB^
	MGFY	84.01 ± 0.03^cA^	74.41 ± 0.02^cB^
	FGFY	132.62 ± 0.02^bA^	128.89 ± 0.01^bB^

*Note*: Yogurts codes are shown in Table 1. Analyses were performed in triplicate. Values are means ± SD. Small and capital letters indicate a significant difference in the columns (the difference between the samples in a day of storage) and the rows (difference of one sample during storage) at level of 5%.

The DPPH radical scavenging activity (IC 50%) of yogurt samples was evaluated on the first and 21st days of storage. Both fortified yogurt samples had significantly higher DPPH radical scavenging activities compared to the control sample, indicating the more stability of grape pomace extract and flaxseed oil combination within the yogurt. The highest antioxidant activity was related to the MGFY on the first day of storage. Moreover, the antioxidant activity of MGFY sample was found to be significantly higher than FGFY, which related to protection effect of maltodextrin and gum tragacanth as wall materials of microcapsules on GP and FO and consequently higher phenolic content in the yogurt (Moghadam et al., 2021).

### 3.8 In vitro gastrointestinal digestion

Phenolic compounds are hydrolyzed after ingestion and convert to metabolites with low antioxidant activity. Microencapsulation can preserve the phenolic compounds and in consequence, the antioxidant activity of food components (Gris et al., 2021). The levels of TPC and antioxidant activity for fortified and control yogurt samples during in vitro gastrointestinal digestion are presented in Table 5. In the gastric and intestinal stages, significant (*p* < .05) higher levels of TPC and antioxidant activity (IC 50) were observed in yogurt samples fortified with combination of GP and FO in microencapsulated or free form, compared to control yogurt. TPC stability and antioxidant activity in gastric and intestinal stages for MGFY was also significantly higher than FGFY, which might be related to protective effect of capsule used on phenolic and antioxidant compounds during passage through the gastrointestinal system (Yadav et al., 2018). Similar results were reported for yogurts fortified with microencapsulated vitamin D and yerba meta extract (Gris et al., 2021; Khan et al., 2020). On the other hand, and as shown in Table 5, the amount of TPC release in intestinal stage was significantly higher than gastric stage (*p* < .05). In this context, Ydjedd et al. (2017) reported higher release of TPC in intestinal phase compared to gastric phase for encapsulated carob pulp extract, due to the protective effect of capsule on phenolic compounds against the changes in digestion conditions including pH variation and type of enzymes.

**TABLE 5:** Release characteristics of yogurts.

	Yogurts	Gastric stage	Intestinal stage
Total release content of phenolic compounds (%)	CY	8.88 ± 0.05^cB^	10.37 ± 0.15^cA^
	MGFY	17.71 ± 0.07^aB^	21.92 ± 0.09^aA^
	FGFY	14.77 ± 0.08^bB^	19.11 ± 0.05^bA^
DPPH radical scavenging activity (IC_50_)	CY	215.81 ± 0.05^aA^	193.99 ± 0.03^aB^
	MGFY	137.63 ± 0.09^cA^	95.55 ± 0.13^cB^
	FGFY	143.12 ± 0.11^bA^	122.42 ± 0.07^bB^

*Note*: Yogurts codes are shown in Table 1. Analyses were performed in triplicate. Values are means ± SD. Small and capital letters indicate a significant difference in the columns (the difference between the samples in a day of storage) and the rows (difference of one sample during storage) at level of 5%.

The TPC and antioxidant activity in the intestinal stage was significantly higher than gastric stage (*p* < .05), which might be due to the pH of the environment. After gastric digestion, the release of phenolics increased due to the change in pH from acidic to alkaline. As in moderate alkaline condition of intestine, the phenolic release increases due to the separation of a proton from the hydroxyl groups of the microcapsules (Zygmantaitė et al., 2021).

### 3.9 Sensory evaluation

The sensory properties determine the acceptance rate of a food by consumers and in consequence the decision of consumers is linked to some characteristics of foods, that is, appearance, color, consistency, odor, and flavor (Pereira et al., 2021).

Sensory properties of control and fortified yogurts are presented in Table 6. The control yogurt had the highest sensory score among the yogurt samples. However, the MGFY received similar scores for all sensory characteristics as CY, statistically. Therefore, it can be concluded that yogurt manufactured using encapsulated GP and FO as bioactive compounds can be preferred by consumers as much as control yogurt. Similar trend was determined by Comunian et al. (2017) for developing a functional yogurt containing free and encapsulated echium oil.

**TABLE 6:** Sensory properties of yogurts during storage.

	Yogurts	1st day	7th day	14th day	21st day
Appearance and Color	CY	5.00 ± 0.00^aA^	5.00 ± 0.00^aA^	4.94 ± 0.06^aB^	4.85 ± 0.11^aB^
	MGFY	5.00 ± 0.00^aA^	4.96 ± 0.05^aAB^	4.92 ± 0.05^aB^	4.83 ± 0.10^aC^
	FGFY	4.77 ± 0.06^bA^	4.71 ± 0.05^bB^	4.65 ± 0.05^bC^	4.58 ± 0.05^bD^
Consistency (perceived with mouth)	CY	5.00 ± 0.00^aA^	4.84 ± 0.11^aB^	4.76 ± 0.05^aC^	4.64 ± 0.05^aD^
	MGFY	5.00 ± 0.00^aA^	4.86 ± 0.05^aB^	4.77 ± 0.05^aC^	4.66 ± 0.11^aD^
	FGFY	5.00 ± 0.00^aA^	4.89 ± 0.05^aB^	4.79 ± 0.09^aC^	4.67 ± 0.11^aD^
Consistency (perceived with spoon)	CY	5.00 ± 0.00^aA^	4.91 ± 0.05^aAB^	4.81 ± 0.11^aB^	4.62 ± 0.05^aC^
	MGFY	5.00 ± 0.00^aA^	4.94 ± 0.05^aAB^	4.83 ± 0.09^aB^	4.65 ± 0.03^aC^
	FGFY	5.00 ± 0.00^aA^	4.94 ± 0.05^aAB^	4.86 ± 0.09^aB^	4.69 ± 0.02^aC^
Odor and flavor	CY	5.00 ± 0.00^aA^	4.94 ± 0.06^aAB^	4.93 ± 0.06^aAB^	4.84 ± 0.05^aB^
	MGFY	4.97 ± 0.06^aA^	4.92 ± 0.05^aAB^	4.90 ± 0.11^aAB^	4.78 ± 0.11^aB^
	FGFY	4.86 ± 0.06^bA^	4.73 ± 0.05^bB^	4.60 ± 0.05^bC^	4.51 ± 0.11^bD^
Overall acceptance	CY	20.00 ± 0.00^aA^	19.69 ± 0.12^aB^	19.44 ± 0.14^aBC^	18.95 ± 0.12^aC^
	MGFY	19.97 ± 0.12^aA^	19.68 ± 0.14^aB^	19.42 ± 0.12^aBC^	18.92 ± 0.17^aC^
	FGFY	19.63 ± 0.09^bA^	19.27 ± 0.06^bB^	18.90 ± 0.22^bC^	18.45 ± 0.23^bD^

*Note*: Yogurts codes are shown in Table 1. Analyses were performed in triplicate. Values are means ± SD. Small and capital letters indicate a significant difference in the columns (the difference between the samples in a day of storage) and the rows (difference of one sample during storage) at level of 5%.

Using mixture of GP and FO in free form for the fortification of stirred yogurt exhibited significant changes in the sensory properties of the final product (*p* < .05) and led to significant decrease in the appearance and color, odor, and flavor and overall acceptability of FGFY compared to CY and MGFY. The presence of free oil and its excessive acidification can affect the odor and flavor of FGFY, negatively (Baba et al., 2018; Zheng et al., 2022). Moreover, the presence of various forms of fruits, that is, pulp, pomace, or powder in formulation of yogurt can increase the acidic character and result in the decrease in the odor and taste of the final product (Bianchini et al., 2020). In a related study, Ghorbanzade et al. (2017) reported that yogurt fortified with nano‐encapsulated fish oil had significantly higher color, taste, and aroma scores than yogurt produced using free form of fish oil. Similarly, significantly lower scores of appearance, odor, taste, and overall acceptance were reported for yogurts fortified with grape pomace obtained from different cultivars compared with the control one (Marchiani et al., 2016).

On the other hand, storage time had significant negative effects on all sensory properties of control and fortified yogurt samples. These findings are generally in accordance with those of previous reports that found the undesirable effect of increasing storage time on the sensory properties of the yogurt samples (Almasi et al., 2021; Baba et al., 2018; Varedesara et al., 2021).

## 4 CONCLUSIONS

Production of functional fermented products such as yogurt is a challenging process due to the metabolic activity of the involved microorganisms on food components. GP and FO are rich sources of bioactive compounds. Fermentation process for yogurt manufacture can affect these compounds and their biological activity. In current study, the combination of these two bioactive compounds (GP and FO) in microencapsulated and free forms was used for manufacturing of stirred yogurt and the quality characteristics of the resulted products were investigated during 21 days of storage. Incorporation of co‐microencapsulated GP and FO for manufacturing of functional stirred yogurt exhibited positive results, especially in terms of TPC and antioxidant capacity of the final product. MGFY showed sensory properties similar to CY, with a significant increase in water holding capacity, viscosity, phenolic, and antioxidant compounds, and a decrease in syneresis rate, which is a crucial parameter in yogurt manufacturing. Furthermore, MGFY showed higher bio‐accessibility for total phenolic compounds and antioxidant activity than FGFY after in vitro digestion, indicating that combination of maltodextrin and gum tragacanth as wall materials for microencapsulation procedure has proper protective properties against in vitro digestion. Given the obtained results, GP and FO as potential bioactive compounds with antioxidant capacity can be used in microencapsulated form for developing functional yogurt in order to promote the consumers' health.

## AUTHOR CONTRIBUTIONS

**Manaf Saberi:** Formal analysis (equal); investigation (equal). **Solmaz Saremnezhad:** Data curation (equal); methodology (equal); supervision (equal). **Mostafa Soltani:** Conceptualization (equal); methodology (equal); visualization (equal); writing – original draft (equal). **Alireza Faraji:** Software (equal); validation (equal); writing – review and editing (equal).

## CONFLICT OF INTEREST STATEMENT

The authors declare that they do not have any conflict of interest.

## ETHICAL APPROVAL

This study does not involve any human or animal testing.

## INFORMED CONSENT

Written informed consent was obtained from all study participants.

## Data Availability

The data that support the findings of this study are available from the corresponding author upon reasonable request.
